# Delivery of phosphatidylethanolamine blunts stress in hepatoma cells exposed to elevated palmitate by targeting the endoplasmic reticulum

**DOI:** 10.1038/s41420-020-0241-z

**Published:** 2020-02-18

**Authors:** Marcus Trentzsch, Eugene Nyamugenda, Tiffany K. Miles, Haven Griffin, Susan Russell, Brian Koss, Kimberly A. Cooney, Kevin D. Phelan, Alan J. Tackett, Srividhya Iyer, Gunnar Boysen, Giulia Baldini

**Affiliations:** 1 Department of Biochemistry and Molecular Biology, University of Arkansas for Medical Sciences, Little Rock, AR USA; 2 Department of Neurobiology & Developmental Sciences, University of Arkansas for Medical Sciences, Little Rock, AR USA; 3 Division of Endocrinology and Metabolism, Center for Osteoporosis and Metabolic Bone Diseases, University of Arkansas for Medical Sciences, Little Rock, AR USA; 4 Department of Environmental and Occupational Health, University of Arkansas for Medical Sciences, Little Rock, AR USA

**Keywords:** Apoptosis, Cell death

## Abstract

Genetic obesity increases in liver phosphatidylcholine (PC)/phosphatidylethanolamine (PE) ratio, inducing endoplasmic reticulum (ER) stress without concomitant increase of ER chaperones. Here, it is found that exposing mice to a palm oil-based high fat (HF) diet induced obesity, loss of liver PE, and loss of the ER chaperone Grp78/BiP in pericentral hepatocytes. In Hepa1–6 cells treated with elevated concentration of palmitate to model lipid stress, Grp78/BiP mRNA was increased, indicating onset of stress-induced Unfolded Protein Response (UPR), but Grp78/BiP protein abundance was nevertheless decreased. Exposure to elevated palmitate also induced in hepatoma cells decreased membrane glycosylation, nuclear translocation of pro-apoptotic C/EBP-homologous-protein-10 (CHOP), expansion of ER-derived quality control compartment (ERQC), loss of mitochondrial membrane potential (MMP), and decreased oxidative phosphorylation. When PE was delivered to Hepa1–6 cells exposed to elevated palmitate, effects by elevated palmitate to decrease Grp78/BiP protein abundance and suppress membrane glycosylation were blunted. Delivery of PE to Hepa1–6 cells treated with elevated palmitate also blunted expansion of ERQC, decreased nuclear translocation of CHOP and lowered abundance of reactive oxygen species (ROS). Instead, delivery of the chemical chaperone 4-phenyl-butyrate (PBA) to Hepa1–6 cells treated with elevated palmitate, while increasing abundance of Grp78/BiP protein and restoring membrane glycosylation, also increased ERQC, expression and nuclear translocation of CHOP, non-mitochondrial oxygen consumption, and generation of ROS. Data indicate that delivery of PE to hepatoma cells under lipid stress recovers cell function by targeting the secretory pathway and by blunting pro-apoptotic branches of the UPR.

## Introduction

An alarming health issue is the prevalence of obesity and its complications in developed countries^1^. In humans, the most frequent cause of obesity is the combination of overfeeding and decreased physical activity^2^. Hypercaloric diets where the excess calories are derived from saturated fat, trans-fat, and refined-grain foods, are associated with weight gain^2–4^. Increased caloric intake eventually exceeds the capacity of the body to store the excess of lipids in adipose tissue, thereby inducing ectopic fat deposition and increasing free fatty acid (FFAs) in the bloodstream. These events contribute to induce liver tissue injury by promoting endoplasmic reticulum (ER) stress and the appearance of nonalcoholic fatty liver (NAFL)^5,6^. NAFL is a positive predictor of obesity complications, such as atherosclerosis, insulin resistance, and type 2 diabetes^5,7^. A well-studied ER response to stress derived from an increased load of misfolded proteins is the unfolded protein response (UPR), a pathway where X-box binding protein 1 (XBP1) is spliced to the active transcription factor XBP1s, which would then promote synthesis of ER chaperones to restore ER homeostasis^8,9^. However, in the liver of the genetically obese Lep−/− mice, abundance of ER chaperones does not appear to be increased^10^. Importantly, in humans, NAFL does not alter abundance of XBP1s protein in liver^11^. Thus, lipid stress may not activate classical UPR with expansion of ER chaperones. In genetic obesity originated from leptin deficiency, de novo lipid synthesis in liver has been found to increase phosphatidylcholine (PC) to phosphatidylethanolamine (PE) ratio in the endoplasmic reticulum (ER) membrane^10^ and to induce ER stress by inhibiting sarco/endoplasmic reticulum calcium ATPase (SERCA) activity. Mitofusin 2 deficiency in mice also alters phospholipid composition, by inducing defective transfer of phosphatidylserine from ER to mitochondria to synthesize PE and liver disease^12^. Exposure to a lard-based HF diet induces changes of liver phospholipid composition including lowering PE abundance^13^. Here, we asked whether obesity by exposure to palm oil based HF diet has also effects to decrease liver PE abundance and to promote ER stress. We also asked whether delivery of PE to hepatoma cells exposed to elevated palmitate counteracts adverse effects by lipid stress and restores function of the secretory pathway.

## Results

### In mice exposed to a palm oil-based HF diet, onset of hepatosteatosis is paralleled by loss of Grp78/Bip in pericentral hepatocytes

**Fig. 1:**
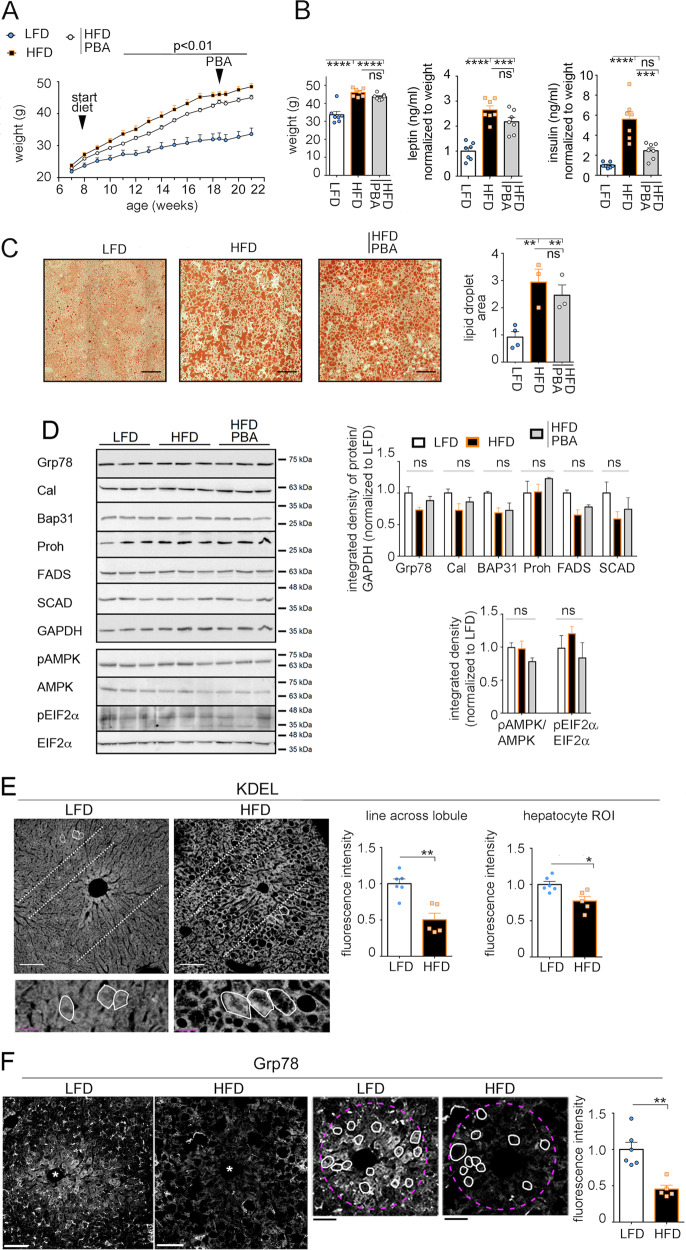
Exposure to a palm oil-based HF diet induces hepatosteatosis without expansion of ER chaperones and loss of Grp78/Bip in pericentral hepatocytes. **a**, **b** Weight of mice fed for 14 weeks with either 10% fat diet (LFD), or 45% palm oil HF diet (HFD). A group of mice fed with 45% HF diet were treated with PBA in the last 2 weeks of treatment (*n* = 7 mice per condition, values are mean ± SEM) (**a**). Weight, and the ratios serum leptin/weight and insulin/weight of the same groups of mice as in **a** (*n* = 7 mice per condition, values are mean ± SEM) (**b**). Statistical analysis is done by one-way Anova; ns, not significant, *p < 0.05, ***p < 0.001, ****p < 0.0001. **c** Lipid droplets in liver sections are stained as described under “Materials and methods” section. The fraction of the total ROI area with lipid droplets is expressed as the ratio—“area of lipid droplets”/total area of ROI—(*n* = 4 mice on lean diet, *n* = 3 mice on 45% HF diet, *n* = 3 mice on 45% HF + PBA, values are mean ± SEM). Black scale bars, 200 μm. Statistical analysis is done by one-way Anova; **p < 0.01. **d** Western blot analysis of liver homogenates (*n* = 3 mice per condition, each lane of the SDS/PAGE gel is loaded with 20 μL of liver homogenate containing 2 μg/μL protein. GAPDH glyceraldehyde-3-phosphate dehydrogenase, Grp78 Grp78/BiP, Cal calreticulin, Bap31 B-cell receptor-associated protein 31, Proh prohibitin, FADS fatty acid desaturase1, SCD1 stearoyl-CoA desaturase-1), Glyceraldehyde 3-phosphate dehydrogenase, AMPK 5′ AMP-activated protein kinase, pAMPK phosphorylated AMPK, eIF2α eukaryotic initiation factor 2α, peIF2α phosphorylated eIF2α. In the graph, values are mean ± SEM. **e** Immunofluorescence microscopy of formaldehyde-fixed liver sections derived from mice fed for 14 weeks with 10% LFD and 45% palm oil HFD (*n* = 5 mice per condition). Sections are immunostained with antibody against KDEL. To measure KDEL abundance across the hepatic lobule, the sum of fluorescence intensities of KDEL immunostaining was calculated by using the segment analysis tool of ImageJ. Each symbol is derived from data obtained from three lines (as the representative dashed white lines shown in **e**) per image and three images per mouse. To measure single-cell KDEL abundance, the average raw fluorescence intensity of KDEL in hepatocyte is derived from 30 recognizable hepatocytes per mouse (as those within white solid lines). In the graph, each symbol represents the average hepatocyte KDEL fluorescence intensity per mouse (*n* = 5 mice per condition). Data are normalized to control mice fed the LF diet. White scale bar, 100 μm; magenta scale bar, 25 μm. Statistical analysis is done by Student’s *t*-test, *p < 0.05, **p < 0.01. **f** Liver sections derived as in **e** are immunostained with antibody against Grp78/BiP. The dashed magenta line draws a circle around the pericentral area of the lobule where fluorescence intensity of Grp78/BiP immunostaining is measured. The average raw fluorescence intensity of Grp78/BiP immunostaining in hepatocyte is derived from 20 morphologically recognizable hepatocytes per mouse (within white solid lines in **f**. In the graph, each symbol represents the average hepatocyte Grp78/BiP fluorescence intensity per mouse (*n* = 5 mice per condition). Images of livers from HFD and LFD-treated mice are shown at higher magnification and with increased brightness. White scale bar, 100 μm; black scale bar, 25 μm; white asterisk shows the central vein. Statistical analysis is done by Student’s *t*-test; *p < 0.05, **p < 0.01.

**Fig. 2:**
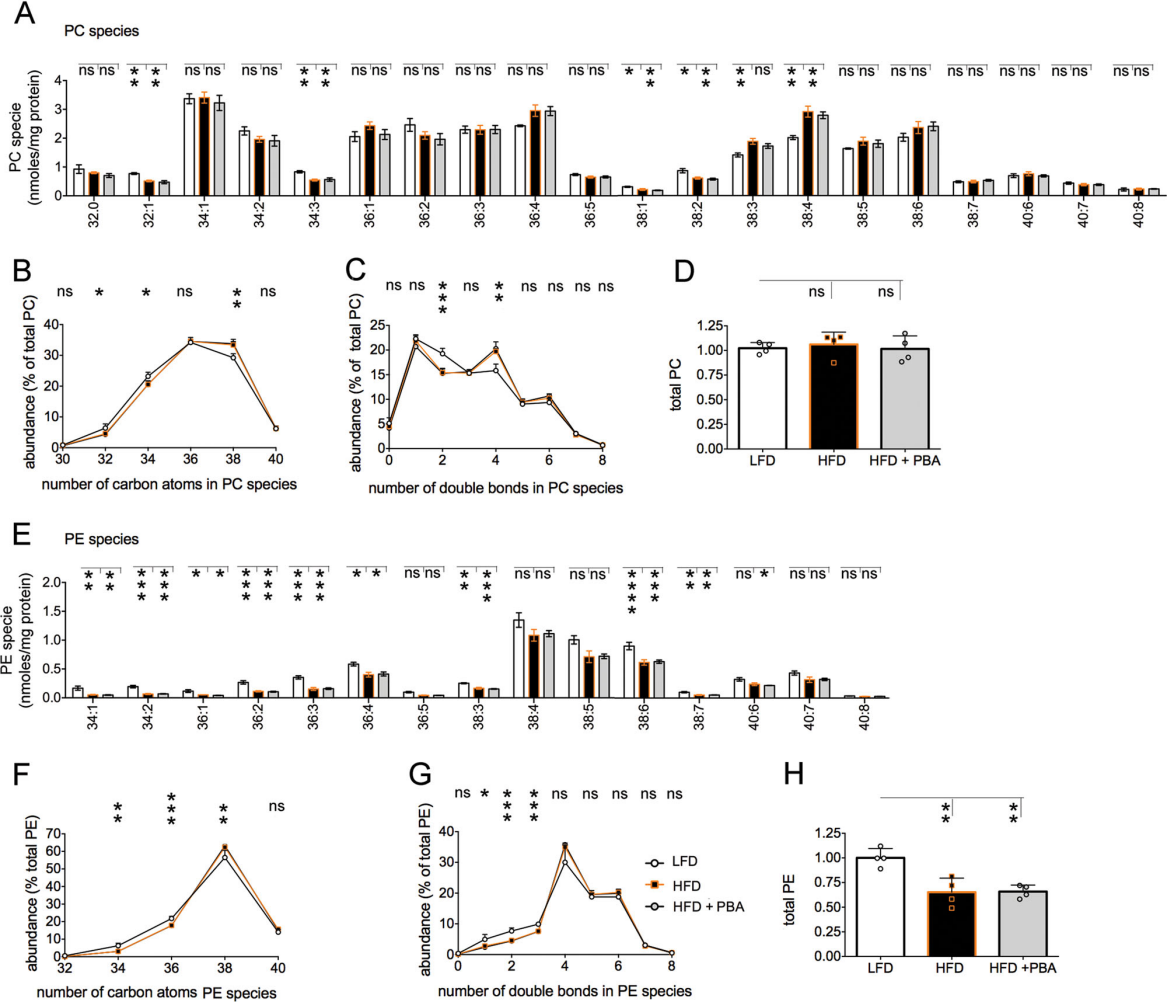
Mice treated with palm oil-based HF diet have reduced abundance of PE in liver. **a**–**d** Abundance of individual PC species in livers derived from the same mice as in Fig. 1, treated with LF diet (LFD, *n* = 4 mice), palm oil HF diet (HFD, *n* = 4 mice), and HFD and PBA (*n* = 4 mice) is expressed as nmoles/mg protein (**a**). PC species are grouped by the number of acyl chain carbon atoms and their relative abundance (nmoles/mg protein) is expressed as percent of total liver PC (nmoles/mg protein) for each condition (**b**); PC species are grouped by the number of double bonds of the two fatty acyl groups combined and their relative abundance (nmoles/mg protein) is expressed as percent of total liver PC (nmoles/mg protein) for each condition (**c**); sum of abundance of each PC species (nmoles/mg protein), is normalized to that of mice treated LFD D). **e**–**h** Abundance of individual PE species in liver from mice treated with LFD and HFD (**e**). PE species are grouped by the number of acyl chain carbon atoms and their relative abundance is expressed as percent of total liver PE (**f**); PE species are grouped by the number of double bonds of the two fatty acyl groups combined and their relative abundance is expressed as percent of total liver PE (**g**); sum of abundance of each PE species is normalized to that of mice treated LFD (**h**). Values are mean ± SD. Statistical analysis is done by one-way Anova; ns not significant, *p < 0.05, **p < 0.01, ***p < 0.001, ****p < 0.0001.

A HF diet with 45% Kcal fat from lard has been used to study effects of lipid stress in rodents^14–16^. We have here modified this diet by substituting lard with palm oil^13^, which reflect more closely the lipid composition of human diets^17–19^. Mice were treated with LF and palm oil-based HF diet for a total of 14 weeks. It has been reported that oral administration of a chemical chaperone, 4-phenyl-butyrate (PBA), to mice with leptin deficiency normalizes metabolic parameters and reverts fatty liver disease^20^. To study whether PBA blunts adverse effects by HF diet exposure, a group of mice were given the chemical chaperone in the drinking water (~15 mg of PBA/day) in the last 2.5 weeks of HF diet treatment. Mice treated with HF diet, with and without PBA treatment, had increased weight as compared to that of mice treated with LF diet (Fig. 1a, b). Mice treated with HF diet had increased level of the ratios circulating leptin/body weight and circulating insulin/body weight as compared that of mice treated with LF diet. PBA treatment decreased the ratio insulin/body weight to a similar value as that of mice treated with LF diet, indicating that the chemical chaperone alleviates insulin resistance in obese mice. Administration of the HF diet increased liver lipid content detected by Oil Red staining, and this effect was not reverted by the PBA treatment (Fig. 1c). In the liver of mice treated with the HF diet with and without PBA treatment, Western blot analysis did not detect changes in abundance of ER proteins or increased phosphorylation of eukaryotic initiation factor-2α (eIF2α), a component of the UPR^21–26^ (Fig. 1d). As division of labor takes place in the mammalian liver^27^, immunofluorescence microscopy of the hepatic lobule may better detect changes in tissue protein abundance upon exposure to HF diet. Livers were derived from another cohort of mice, also treated with LF and HF diet, but where tissues were harvested after trans-cardiac fixative perfusion. KDEL is a four amino acid sequence that allows retention of ER proteins in the ER^28^. In the liver of mice treated with LF diet, KDEL immunostaining showed plates of hepatocytes radiating from the central vein (Fig. 1e). Exposure to HF diet changed the overall morphology of the hepatic lobule, with decreased cell density and increased hepatocyte volume. In mice exposed to HF diet, the overall intensity of KDEL immunostaining across the hepatic lobule (along the dashed white lines) and in hepatocytes (within white perimeters) was decreased as compared of that from mice treated with LF diet. Grp78/Bip, an abundant ER chaperone, appeared to be expressed at highest levels in pericentral hepatocytes (within circles outlined by magenta dashed line, Fig. 2f). By single cell analysis, abundance of GrP78/BiP protein in pericentral hepatocytes was decreased upon exposure to HF diet.

### Mice fed palm oil-based HF diet have loss of PE in liver

We have recently found that exposure to the lard-based HF diet induces loss of PE but not of PC, in liver^13^. Here, we asked whether exposure to palm oil-based HF diet altered liver PC and PE abundance and composition. In the liver of mice fed for 14 weeks with the HF diet, with or without PBA treatment, some PC species, such as those with 32:1, 34:3, 38:1, and 38:2 acyl chains were decreased, while other PC species such as those with 38:4 acyl chains were instead increased (Fig. 2a). The most abundant PC species in liver were those with 36 and 38-carbon atom acyl chains and treatment with HF diet, with or without PBA, further increased relative abundance of PC species bearing 38-carbon atom acyl chains and decreased that of PC species with shorter chains of 32 and 34 carbon atoms (Fig. 2b). Exposure to HF diet, with or without PBA treatment, decreased the relative abundance of total PC species bearing two double bonds while increasing that of PC species with 4 double bonds (Fig. 2c). Exposure to the HF diet, with or without the PBA treatment, left unchanged total abundance of liver PC (Fig. 2d). In the liver of mice fed HF diet, with and without PBA treatment, abundance of most PE species was decreased (Fig. 2e). Exposure to the HF diet also induced a shift of acyl chain length, with a relative increase of PE species with long chains of 38 carbon atoms and a decrease of PE species bearing shorter 34 and 36 carbon atoms (Fig. 2f), as well as decreased relative abundance of species with one, two, and three double bonds (Fig. 2g). Overall, exposure to HF diet induced loss of total PE abundance and this effect was not reverted by PBA treatment (Fig. 2h).

### Delivery of PE to Hepa1-6 cells exposed to elevated palmitate increases by post-transcriptional mechanisms Grp78/BiP expression and blunts activation of CHOP

**Fig. 3:**
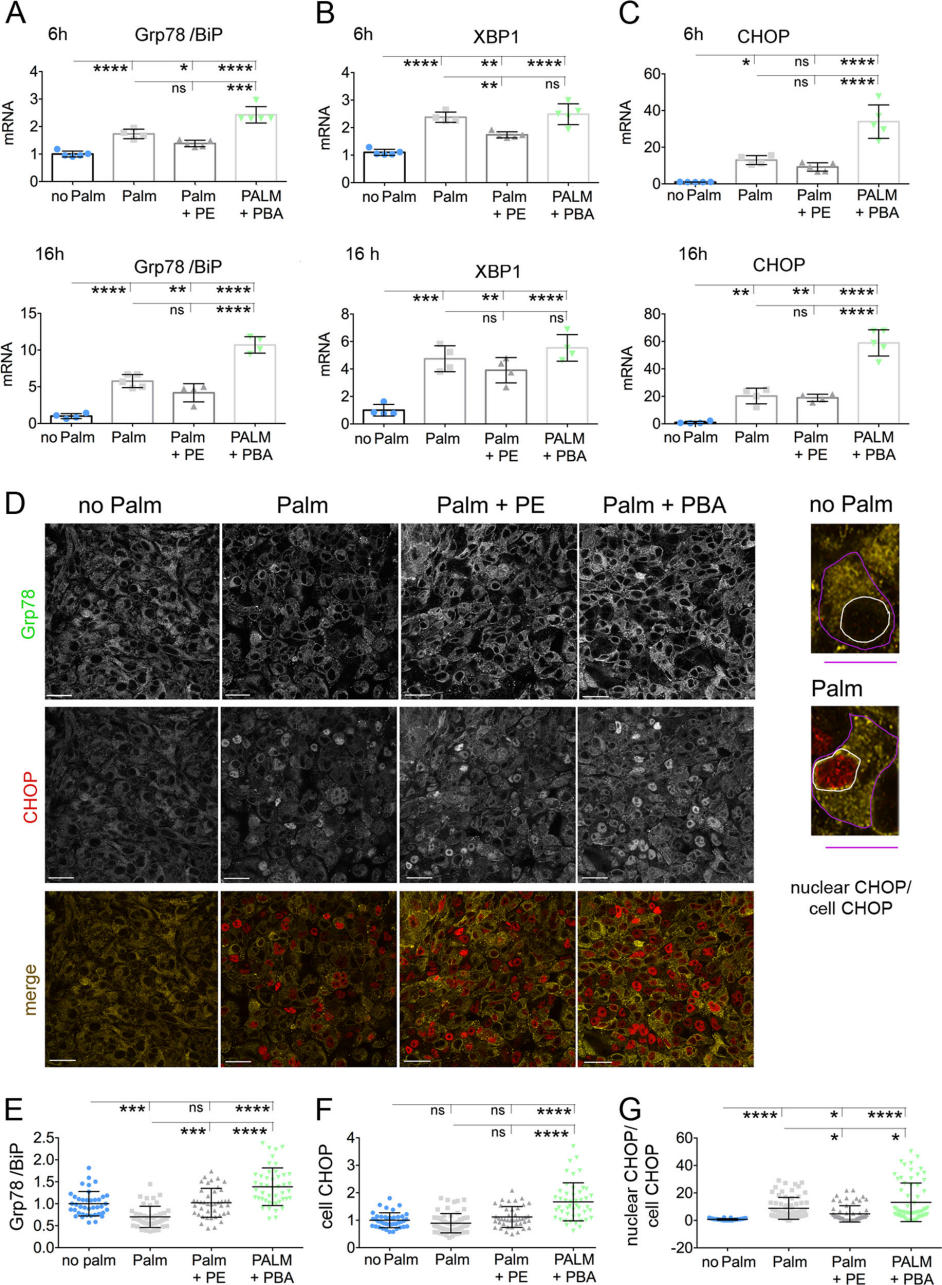
PE and PBA, delivered to Hepa1–6 cells exposed to lipid stress, have different effects to increase expression of Grp78/Bip and to promote activation of CHOP. **a**–**c** Abundance of Grp78/BiP (**a**), Xbp1s (**b**), and CHOP mRNA (**c**) in samples derived from Hepa1–6 cells treated with: no additions; 200 μM palmitate; 200 μM palmitate and 25 μM PE, 200 μM palmitate and 2 mM PBA for 6 h and 16 h, respectively (samples are derived from *n* = 5 plates of Hepa1–6 cells per condition). **d**–**g** Immunofluorescence analysis of Grp78/BiP and CHOP in samples derived from cells treated as in **a** for 16 h. CHOP translocation to the nucleus (nuclear CHOP) is monitored by the ratio-pixel intensity of CHOP immunofluorescence in nucleus (pixel intensity in area enclosed by white line)/pixel intensity of CHOP immunofluorescence in cell (pixel intensity in area enclosed by magenta line) (**d**, **g**). Data are representative of three independent experiments and are expressed as mean ± SD. White scale bars, 50 µm; magenta scale bars, 25 µm. Statistical analysis is done by one-way Anova; ns, not significant, *p < 0.05, **p < 0.01, ***p < 0.001, ****p < 0.0001.

Most of the triacylglycerol that accumulates in the liver of obese individuals is contributed by elevated circulating FFAs^29,30^. Exposure to HF diet induces loss of Grp78/BiP in pericentral hepatocytes. To determine whether elevated extracellular FFAs is sufficient to induce loss of Grp78/BiP protein, we treated a liver cell line derived from murine hepatoma, Hepa1–6 cells, with elevated palmitate coupled to BSA. When Hepa1–6 cells were incubated with 0.2 mM palmitate for 6 and 16 h, Grp78/BiP mRNA was increased (Fig. 3a), while at 16 h abundance of Grp78/BiP protein was instead decreased (Fig. 3d, e). Thus, exposure to the elevated palmitate induces transcriptional upregulation of ER chaperone expression, which is then not executed at the protein level, with instead loss of Grp78/BiP protein. Classical UPR induced by increased load of misfolded protein increases splicing of X-box binding protein 1 (XBP1) to XBP1s, a transcription factor that promotes expression of ER chaperones including that of Grp78/BiP^31^. When Hepa1–6 cells were incubated with 0.2 mM palmitate for 6 and 16 h, XBP1s mRNA was increased, indicating transcriptional upregulation of the UPR (Fig. 3b). Another UPR component is the transcription factor C/EBP homologous protein (CHOP), which is activated under conditions of ER stress and promotes cell death^32^. When Hepa1–6 cells were incubated with 0.2 mM palmitate for 6 and 16 h, CHOP mRNA was increased (Fig. 3c), but the total abundance of CHOP protein nevertheless remained unchanged as compared to that of cells not exposed to the elevated fatty acid (Fig. 3d, f). Instead, exposure to elevated palmitate increased the ratio—nuclear CHOP/total cell CHOP—(Fig. 3g), thus indicating that exposure to the elevated fatty acid induces translocation of the transcription factor from the cytoplasm to the nucleus. As PE is reduced in the liver of mice fed lard and palm oil-based HF diets (Fig. 2 and data by Nyamugenda et al.^13^), we asked whether delivery of PE to Hepa1–6 cells treated with the elevated palmitate would change response to lipid stress. When PE was added together with the elevated palmitate to the Hepa1–6 cell medium, Grp78/BiP and XBP1s mRNA abundance was increased as compared to cells kept under control conditions, indicating that the transcriptional execution of the UPR induced by elevated fatty acid is still taking place (Fig. 3a, b). In the palmitate treated cells, PE delivery increased abundance of Grp78/BiP protein to the same level as that of cells not exposed to elevated fatty acid (Fig. 3d, e). Thus, PE delivery to Hepa1–6 cells exposed to elevated palmitate blunts the loss of ER chaperones induced by exposure to elevated fatty acids. In the Hepa1–6 cells treated with both elevated palmitate and PE, CHOP mRNA was expressed at the same elevated level as that of Hepa1–6 cells exposed to excess fatty acid (Fig. 3c). However, in palmitate-exposed Hepa1–6 cells treated with PE, effects by the elevated fatty acid to induce nuclear translocation of CHOP were blunted (Fig. 3g). Thus, delivery of PE modulates post-transcriptionally response to lipid stress by enhancing expression of the ER chaperone Grp78/BiP and by blunting CHOP nuclear translocation. Differently, when Hepa 1-6 cells incubated with elevated palmitate were exposed to PBA, levels of XBP1s mRNA (Fig. 3b) and levels of Grp78/BiP and CHOP mRNA (Fig. 3a, c) and protein (Fig. 3d–f) were increased at the highest level, and so was the extent of CHOP translocation to the nucleus (Fig. 3g). Thus, delivery of PBA modulates transcriptionally and post-transcriptionally response to lipid stress by enhancing expression of the ER chaperone Grp78/BiP and by promoting CHOP activation.

### Delivery of PE to Hepa1–6 cells exposed to elevated palmitate blunts re-organization of the ER towards ER-associated degradation (ERAD)

**Fig. 4:**
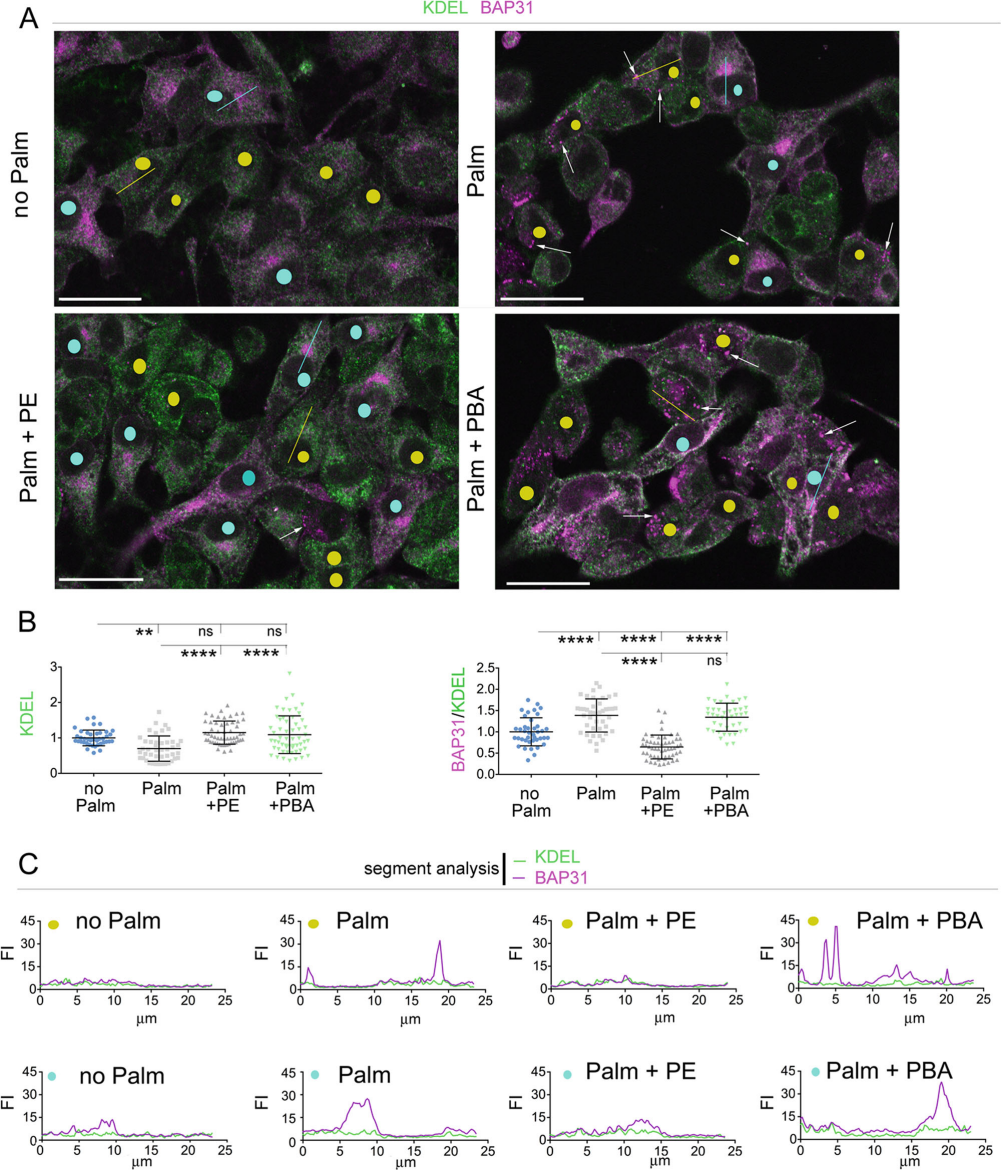
Delivery of PE, but not of PBA, to Hepa 1-7 cells exposed to elevated palmitate blunts re-organization of the ER towards ERAD. **a**–**c** Hepa1–6 cells treated with: no additions; 200 μM palmitate; 200 μM palmitate and 25 μM PE, 200 μM palmitate and 2 mM PBA for 16 h are immunostained with antibodies against KDEL and Bap31. Yellow dots indicate cells with diffuse cytoplasmic staining of Bap31 in peripheral ER; blue dots indicate cells with iuxanuclear accumulation of Bap31 in ERQC; arrows indicate scattered cytoplasmic sites of Bap31 accumulation (cytoplasmic ERQC sites) (**a**). Graphs show fluorescence intensity of KDEL immunostaining and ratio—Bap31 immunofluorescence intensity/KDEL immunofluorescence intensity—(Bap31/KDEL). Data in graphs are normalized to control (no Palm) (**b**). Segment analysis shows pixel intensities of fluorescence immunostaining along the yellow (graphs with yellow dots) and blue lines (graphs with blue dots) drawn in **a** (**c**). Scale bars, 25 μm. Data are expressed as mean ± SD and are representative of three independent experiments. Statistical analysis is done by one-way Anova; ns, not significant, **p < 0.01, ****p < 0.0001.

In response to stress by increased load of unfolded proteins, classical UPR increases both ER chaperones and ERAD to maintain ER homeostasis^31,33^. With this respect, Bap31 functions in ERAD at both early and late stages by interacting with the ER pre-protein translocon Sec61 and with the ER retro-translocon derlin1, respectively^34^. Bap31 moves between the main ER and the juxtanuclear ER-derived quality control compartment (ERQC)^35,36^. We reasoned that an increased ER capacity towards ERAD in Hepa1–6 cells exposed to elevated palmitate might induce increased abundance of Bap31 as well as Bap31 redistribution from the main ER to the ERQC. Bap31 immunostaining appeared in most untreated cells as diffuse cytoplasmic fluorescence similar to that of KDEL (Fig. 4a, cells indicated by yellow dots and Fig. 4c, graph of with yellow dot showing fluorescence intensities of KDEL and Bap31 immunostaining measured by ImageJ segment analysis in a representative cell). Instead, in other Hepa1–6 cells, Bap31 immunostaining appeared to concentrate at the juxtanuclear localization, where KDEL immunostaining did not accumulate (Fig. 4a, cells with blue dots and Fig. 4c, graph with blue dot, segment analysis of a representative cell), thus indicating that ERQC is distinct from main ER. Exposure to elevated palmitate decreased abundance of KDEL and increased the ratio—Bap31 immunofluorescence intensity/KDEL immunofluorescence intensity (Bap31/KDEL) (Fig. 4b). In cells exposed to elevated palmitate that had detectable iuxtanuclear ERQC, accumulation of Bap31 at this site was increased (Fig. 4a, cells and Fig. 4c, graph with blue dots). Moreover, in Hepa1–6 cells exposed to elevated palmitate without detectable ERQC, BAP31 accumulations that did not coincide with increased KDEL appeared as scattered cytoplasmic ERQC sites (Fig. 4a, cells with yellow dots, the arrows indicate scattered cytoplasmic ERQC sites analyzed in graph with yellow dots, Fig. 4c). Thus, in Hepa1–6 cells, exposure to elevated palmitate redistributes BAP31 from peripheral ER to ERQC and forms ERQC sites at cytoplasmic locations. Delivery of PE to Hepa1–6 cells exposed to elevated palmitate increased KDEL immunostaining to the same level as that of cells not exposed to elevated palmitate, thus indicating that ER protein capacity is restored. Moreover, PE delivery to palmitate-treated cells decreased the ratio −Bap31/KDEL- ratio and abundance of Bap31 both at iuxtanuclear and scattered cytoplasmic ERQC, thus indicating that delivery of the phospholipid blunts re-organization of the ER to increase ERQC. Delivery of PBA to Hepa1–6 cells exposed to elevated palmitate also increased KDEL immunostaining to the same level as that of untreated cells, but also elevated the ratio –Bap31/KDEL- and increased abundance of BAP31 at the iuxtanuclear ERQC and at scattered cytoplasmic ERQC sites. Therefore, differently than PE, PBA delivery to palmitate treated cells not only promotes increased ER protein capacity, but also induces a re-organization of the ER that includes expansion of the ERQC.

### Delivery of PE and PBA to Hepa1–6 cells exposed to elevated palmitate rescues membrane glycosylation

**Fig. 5:**
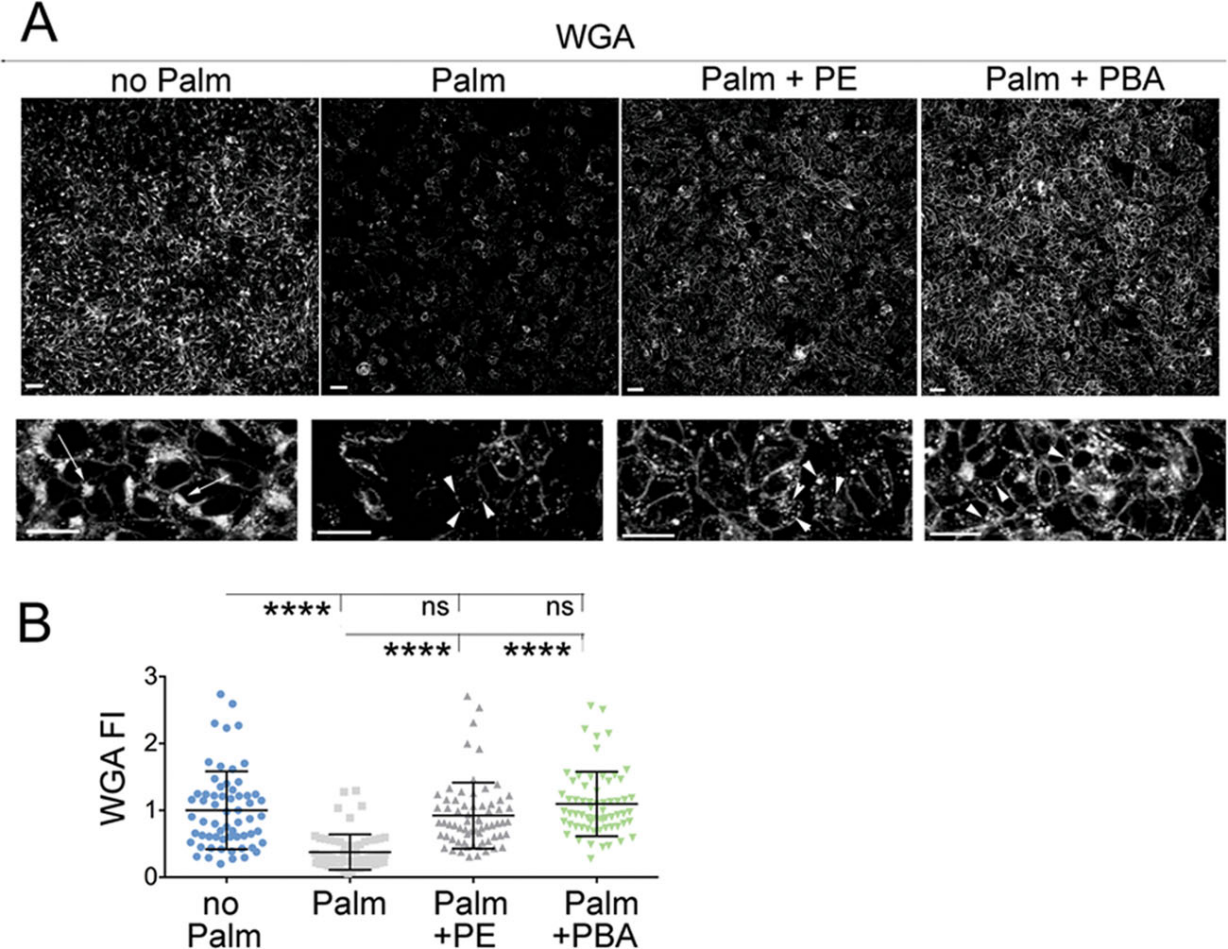
Delivery of both PE and PBA to Hepa1–7 cells exposed to elevated palmitate restores membrane glycosylation. **a** Hepa1–6 cells treated with: no additions; 200 μM palmitate; 200 μM palmitate and 25 μM PE, 200 μM palmitate and 2 mM PBA for 16 h are fixed and stained with wheat germ agglutinin (WGA). White arrows indicate trans-Golgi compartment and white arrowheads indicate fragmented trans-Golgi compartment. Scale bars, 25 μm; Data are expressed as mean ± SD and are representative of three independent experiments. Statistical analysis is done by one-way Anova; ns, not significant, ****p < 0.0001.

Delivery of either PE or PBA to Hepa1–6 exposed to elevated palmitate increases ER protein capacity including that of the chaperone Grp78/BiP. Thus, it is possible that delivery of PE and PBA to cells exposed to elevated palmitate promotes ER and Golgi function, including glycosylation of membrane proteins and sphingolipids^37^. Wheat germ agglutinin (WGA) selectively binds to terminal sialic acid moieties and N-acetylglucosamine found in N-glycans and O-glycans of membranes proteins and in glycosphingolipids (O-GlcNAc)^38–41^. Glycosylated proteins and lipids are abundant at the outer leaflet of the plasma membrane and in the trans-Golgi. In fixed and permeabilized Hepa1–6 cells, fluorescently labeled WGA localized to the plasma membrane and to juxtanuclear trans-Golgi (arrows, Fig. 5). When elevated palmitate was added to cells, cell abundance of membrane glycosylation was reduced. The iuxtanuclear trans-Golgi labeled by WGA virtually disappeared and cytoplasmic WGA staining appeared instead as puncta, indicating trans-Golgi fragmentation. Differently, when either PE or PBA was added to the Hepa1–6 cells incubated with the elevated palmitate, total abundance of membrane glycosylation was restored to a similar level as that of cells incubated in the absence elevated palmitate. However, in palmitate-treated Hepa1–6 cells, fragmentation of the trans-Golgi remained unchanged by PE treatment and was not restored by PBA treatment (arrowheads). Thus, delivery of both PE and PBA restores the glycosylation capacity of the secretory pathway without recovering its normal morphology.

### PE delivery to Hepa1–6 cells exposed to elevated palmitate decreases generation of ROS, but does not restore mitochondrial function

**Fig. 6:**
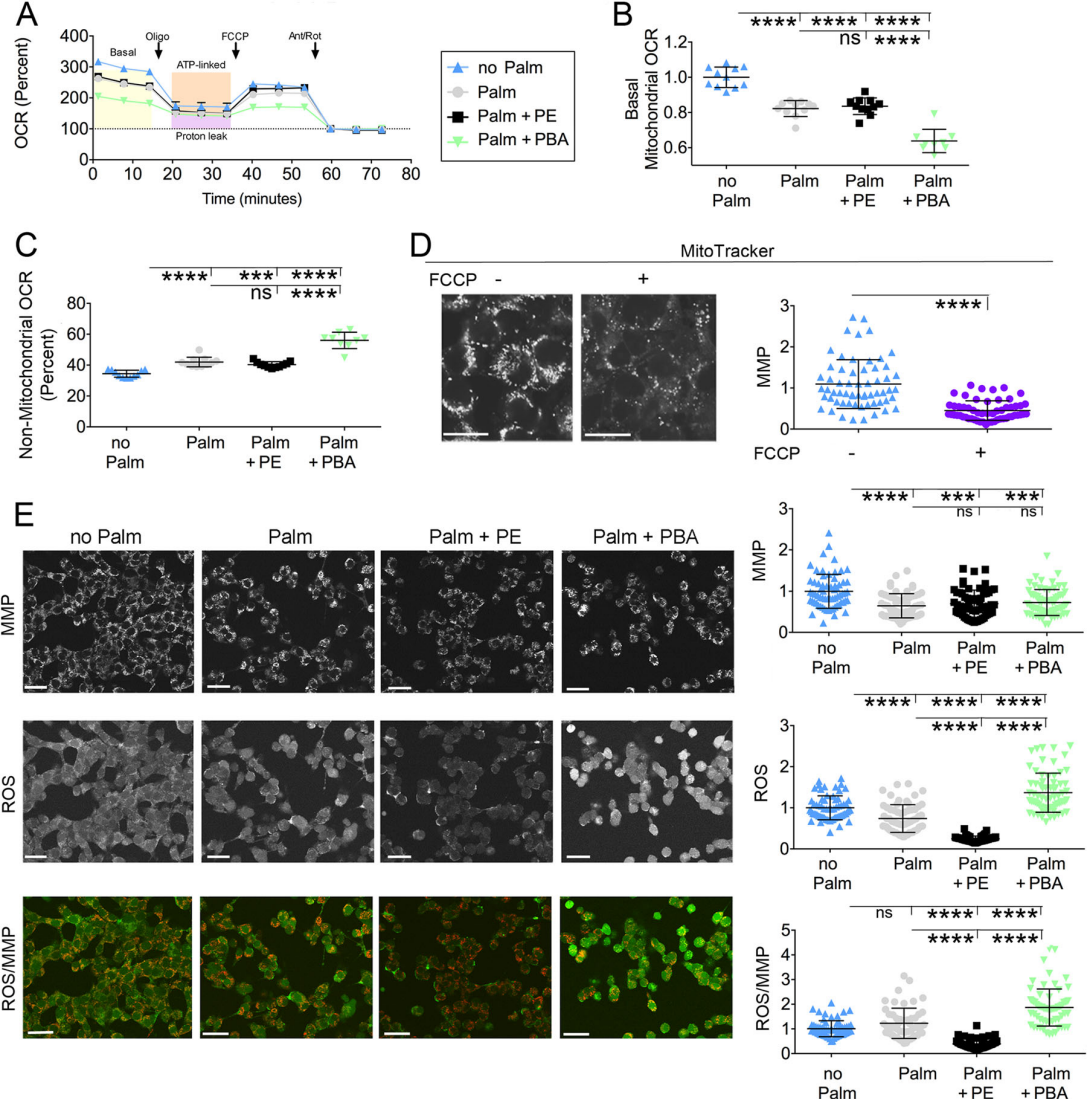
PE and PBA, delivered to Hepa1–6 cells exposed to lipid stress, have different effects to modulate generation of ROS. **a**–**c** OCR is measured in Hepa1–6 cells treated with: no additions; 200 μM palmitate; 200 μM palmitate and 25 μM PE, 200 μM palmitate and 2 mM PBA for 16 h. Arrows in **a** indicate addition to samples of oligomycin (Oligo), antimycin (Ant) and rotenone (Rot). OCR values, normalized per protein content, are expressed as percent of values obtained after addition of Ant and Rot. Data are shown as mean ± SEM (from data derived from *n* = ≥9 wells). Data are representative of two independent experiments (**a**). Basal mitochondrial OCR is derived from data in **a** by calculating the percentage loss of OCR upon addition of oligomycin. Data, normalized to the control (no Palm), are expressed as mean ± SD (**b**). Non-mitochondrial OCR is measured from the OCR of samples treated with of antimycin and rotenone, as indicated in **a**. Data, normalized to the control (no Palm), are expressed as mean ± SD (**c**). Statistical analysis is done by one-way Anova; ns not significant, *p < 0.05, ***p < 0.001, ****p < 0.0001. **d** MitoTracker fluorescence intensity with and without incubation with 1 μM FCCP. Data, normalized to the control (no Palm), are expressed as mean ± SD. Scale Bars, 25 μm. Statistical analysis is done by Student’s *t*-test, ***p < 0.0001. **e** Cells treated as in **a** are incubated with FC-Fluorescein (5 μM) and MitoTracker (200 nM) as described under “Materials and methods” section. Scale bars, 50 μm. Data are expressed as mean ± SD and are representative of three independent experiments. Data in graphs are normalized to control (Hepa-6 cells incubated without palmitate). Statistical analysis is done by one-way Anova; ns not significant, *p < 0.05, **p < 0.01, ***p < 0.001, ****p < 0.0001.

Obese patients and mice with hepatosteatosis have mitochondrial dysfunction with defective oxidative phosphorylation^42–44^. Addition of elevated palmitate to Hepa1–6 cells decreased the rate of oxygen consumption rate (OCR) sensitive to oligomycin, thereby indicating loss of oxidative phosphorylation (Fig. 6a, b) and increased non-mitochondrial OCR (Fig. 6c). In Hepa1–6 cells exposed to elevated palmitate and treated with PE, the rate of oligomycin-sensitive OCR remained decreased and non-mitochondrial OCR remained increased to the same extent as that of cells incubated only with the elevated fatty acid. Delivery of PBA to the Hepa1–6 cells exposed to elevated palmitate further decreased oligomycin-sensitive OCR and instead concomitantly increased non-mitochondrial OCR (Fig. 6c). Thus, in the Hepa1–6 cells exposed to elevated palmitate, delivery of either PE or PBA does not restore mitochondrial function. It is possible that, in the palmitate-treated cells, decreased oxidative phosphorylation is paralleled by decreased ability to generate mitochondrial membrane potential (MMP)^45^. When Hepa1–6 cells were pre-treated with 1 μM FCCP, MitoTracker fluorescence intensity was decreased (Fig. 6d), thus indicating that fluorescence intensity of the dye measures MMP^46^. When Hepa1–6 cells were exposed to elevated palmitate, fluorescence intensity of MitoTracker and of dichlorofluorescin diacetate (DCF), a ROS-sensitive probe^47^, were both reduced so that the ratio ROS/MMP was unchanged (Fig. 6e). Thus, in palmitate-exposed cells, loss of MMP and blunted generation of ROS parallel the decreased in oxidative phosphorylation induced by the elevated fatty acid. When PE was added to the Hepa1–6 cells exposed to elevated palmitate loss of MMP, measured by MitoTracker, again paralleled loss of mitochondrial capacity to function in oxidative phosphorylation, but cellular ROS abundance was further reduced, with decreased ratio ROS/MMP. Thus, PE delivery to hepatoma cells under lipid stress may increase cell capacity to eliminate ROS generated by oxidative phosphorylation. Differently, when Hepa1–6 cells exposed to elevated palmitate were treated with PBA, MMP remained decreased, but the level of ROS being generated was increased. Thus, in palmitate-exposed hepatoma cells, PE limits and PBA enhances the amount of ROS being generated.

## Discussion

We have found that exposure of mice to lard-based HF diet induces loss of liver PE, which takes place even before the onset of obesity^13^. Here, we find that loss of PE takes place also in the liver of obese mice exposed to a palm oil-based HF diet and is concomitant to hepatosteatosis. In human liver, NAFL does not alter protein abundance of XBP1s and CHOP^11^, suggesting that hepatosteatosis may not activate classical UPR. Consistent with that, we find by Western blot analysis that exposure to HF diet does not increase abundance of the chaperones GrP78/BiP and calnexin in murine liver. Instead, by using immunostaining of liver sections, we find for the first time that exposure of mice to the palm oil-based HF diet induces loss of abundance of the ER chaperone GrP78/BiP in pericentral hepatocytes. This effect is replicated in murine hepatoma cells exposed to elevated palmitate. In the hepatoma cells exposed to elevated palmitate, mechanisms of classical UPR to increase cell level of GrP78/BiP and CHOP are taking place at the transcriptional level, but not at the protein level. Delivery of PE to hepatoma cells exposed to the elevated fatty acid restores post-transcriptionally normal abundance of GrP78/BiP protein and function of the secretory pathway. The ER folding machinery functions in connection with the ER protein degradation machinery, which exports for degradation in the cytosol proteins that failed to fold^48^. Delivery of PE to hepatoma cells exposed to elevated palmitate virtually eliminates the expansion of ERQC induced by exposure to the elevated fatty acid. Thus, in hepatoma cells exposed to lipid stress, PE promotes folding capacity of the ER and prevents accumulation of misfolded proteins in ERQC. When ER protein homeostasis is not restored, the UPR initiates apoptotic cell death via up-regulation of CHOP^49^. Here we find that cell exposure to elevated palmitate induces, rather than increased levels of cell CHOP, translocation of the transcription factor to the nucleus, and that this effect is blunted by PE delivery. Thus, in hepatoma cells exposed to elevated palmitate, PE-dependent improvement of ER folding capacity halts CHOP activation. In hepatoma cells exposed to elevated palmitate, oxidative phosphorylation remains decreased when PE is delivered to the cells, suggesting that the treatment is ineffective to resolve mitochondrial stress. Delivery of PBA to hepatoma cells exposed to lipid stress, while promoting increased GrP78/BiP protein abundance and restoring membrane glycosylation capacity, also promotes transcriptionally and post-transcriptionally activation of CHOP. CHOP induces cell death by increasing protein synthesis and by enhancing oxidation in the ER^32,49^. Consistent with these reports, delivery of PBA to hepatoma cells exposed elevated palmitate increases non-mitochondrial oxygen consumption, promotes generation of ROS, and expands the ERQC. Thus, PBA-dependent recovery of ER function may involve increased protein turnover with excessive generation of ROS^50^. We find that, in diet-induced obesity, weight gain and hepatosteatosis were not reversed by treatment with the chemical chaperone PBA. Nevertheless, PBA corrected hyperinsulinemia in mice with HF diet-induced obesity, as previously observed in mice with genetic obesity^20^. Adverse effects of PBA to promote apoptosis and to increase ROS might limit its usefulness to treat hepatosteatosis in vivo. In conclusion, this work indicates delivery of PE as a novel tool to promote hepatic cell recovery from lipid stress. This work opens up the possibility that supplementing diet with PE may be beneficial to improve hepatocyte function in NAFL.

## Materials and methods

### Reagents

Tris-HCl at molecular biology grade was purchased from Promega Corporation (Madison, WI). The 0.9% sodium chloride solution was from Baxter Healthcare Corporation (Deerfield, IL) and sodium phenylbutyrate (PBA) from Santa Cruz Biotechnology (Dallas, TX). Pierce BCA Protein Assay Kit, Pierce Protease Inhibitor Mini tablets (EDTA-free) and 1.5 mL polypropylene safe-lock tubes for lipid extractions as well as the Savant DNA 120 SpeedVac Concentrator were from Thermo Scientific Inc. (Rockford, IL). Heparin (Cat #P87721) was purchased from Braun Medical. (Bethlehem, PA, USA). The Ultra-Sensitive Mouse Insulin ELISA Kit as well as the Mouse Leptin ELISA Kit were from Crystal Chem Inc. (Downers Grove, IL). The MiniCollect serum separation tubes were from Greiner Bio-one (Monroe, NC), and the Teflon pestle tissue homogenizer was purchased from Thomas Scientific (Swedesboro, NJ). The Bioruptor UCD-200 Sonicator was from Diagenode (Denville, NJ). The 300 µl low adsorption amber glass QSertVials with pre-slit PTFE/silicone septa were purchased from Supelco (Bellefonte, PA). Primary antibodies for the following proteins were purchased from Cell Signaling: AMPK (Cat # 2532), pAMPK (Cat # 2535), CHOP (Cat # 5554), eIF2α (Cat # 9722), peIF2α (Cat # 9721 and #3398), GAPDH (Cat # 2118), and SCD1 (Cat # 2438). Primary antibodies for the following proteins were purchased from Abcam: COXIV (Cat #ab16056), FADS1 (Cat #ab126706), Grp78/BiP (Cat #ab21685), and Prohibitin (Cat #ab28172). The antibody against KDEL was purchased from Enzo Life Sciences (Cat#: ADI-SPA-827). Antibodies for BAP31 and calreticulin were purchased from Santa Cruz (Cat #sc-48766) and BD Transduction Laboratories (Cat #612136), respectively. Secondary POD conjugated goat anti-rabbit antibody was purchased from Kirkegaard and Perry Lab (Cat #074-1506). Secondary POD conjugated mouse anti-rabbit antibody (Cat# 211-032-171), Alexa Fluor® 647-conjugated donkey anti-rabbit antibodies (Cat # 711-605-152), Cy3-conjugated goat anti-mouse antibody (Cat#115-165-146), and DyLight 488-conjugated goat anti-rabbit antibodies (Cat # 111-485-144) were purchased from Jackson ImmunoResearch. Wheat Germ Agglutinin conjugated to Alexa Fluor™ 647 (Cat #W32466), MitoTracker™ Red CMXRos (MitoTracker), and Hank’s Balanced Salt Solution (HBSS) were purchased from Thermo Fisher Scientific. The lipid standards 1,2-diheptadecanoyl-sn-glycero-3-phosphoethanolamine (PE C17:0/C17:0, Cat # 830756, CAS # 140219-78-9) and 1,2-diheptadecanoyl-sn-glycero-3-phosphocholine (PC C17:0/C17:0, Cat # 850360, CAS # 70897-27-7) were purchased from Avanti Polar Lipids, Inc. (Alabaster, AL, USA, RRID: SCR_016391). The 2-propanol (i-PrOH) Optima LC/MS grade (Cat # A461-500, CAS # 67-63-0), methyl tert-butyl ether (MTBE) HPLC grade (Cat # AA41839AK, CAS # 1634-04-4), and sodium chloride (Cat # S271-500, CAS # 7647-14-5) were from Fisher Scientific (Fair Lawn, NJ, USA, RRID: SCR_008452). Formic acid (Cat # 33015, CAS # 64-18-6), water (Cat # 34877, CAS # 7732-18-5) and methanol of HPLC grade (Cat # 34860, CAS # 67-56-1), ammonium acetate (Cat # A1542, CAS # 631-61-8), sodium fluoride of BioXtra grade (Cat #: S7920, CAS #: 7681-49-4), L-α-Phosphatidylethanolamine (PE) from egg yolk (Cat # P7943), Oil Red O (Cat # O0625), 2,7-Dichlorofluorescin diacetate (Cat # D6883), carbonyl cyanide 4-(trifluoromethoxy)phenylhydrazone (Cat # C2920), oligomycin A (Cat # 75351), antimycin A (Cat # A8674), rotenone Cat # (R8875), fatty acid-free bovine serum albumin (Cat # A7906), sodium palmitate (Cat # P9767) were purchased from Sigma-Aldrich, Co. (St. Louis, MO). Mouse on Mouse (M.O.M.®) Basic Kit was purchased from Vector laboratories, Cat # BMK-2202). Seahorse XFe96 FluxPak mini (Cat #102601-100), Seahorse XF 1.0 M glucose solution, 50 mL (Cat # 103577-100) Seahorse XF 100 mM pyruvate solution, 50 mL (Cat # 103578-100), Seahorse XF Calibrant Solution 100 mL (103059-000), Seahorse XF base medium, 500 mL (Cat # 103334-100) from Agilent (Santa Clara, CA). Tissue-Tek was purchased from Fisher Healthcare.

### Animals

All animal care protocols have been approved by UAMS Institutional Animal Care and Use Committee AUP #3788. C57BL/6J DIO mice obtained from Jackson Laboratory (Bar Harbor, ME) were housed in polycarbonate cages on a 12-h light-dark cycle at University of Arkansas for Medical Sciences. Diets were purchased from Research Diets, Inc. and administered to mice starting at 8 weeks of age. Mice were administered a palm oil-based HF diet D15012001 (4.70 kcal/g, 45 kcal% fat^13^) and control diet D12450B (3.85 kcal/g, 10 kcal% fat) for 14 weeks. In a separate study 8-week-old mice were treated as follows: (a) one group of mice received the control diet (D12450B); (b) two groups of mice were administered the palm oil-based HF diet D15012001. Two weeks prior to harvesting tissues (week 11.5–14), the drinking water of one group of mice kept on the 45% HF diet was replaced with a solution containing 30 mM PBA. The other two groups of mice treated with control diet and palm oil-based HF diet had 30 mM NaCl in their drinking water.

### Harvesting of liver for Western blot analysis, extraction and analysis of PC and PE

Prior to euthanasia by CO_2_ asphyxiation and decapitation by guillotine as previously described, mice were fasted for 4 h, and blood was collected retro-orbitally. The liver was collected as a ~2 mm wide blocks. Serum and tissues were snap-frozen in liquid nitrogen and stored at −80 °C. Insulin and leptin levels in the serum, were measured by using the Ultra-Sensitive Mouse Insulin ELISA Kit and the Mouse Leptin ELISA Kit, respectively. Extraction of PC and PE from liver was carried out as described previously^13^.

### Oil red O staining

Liver pieces (20–30 mg) were flash frozen in liquid N_2_ after harvesting. Frozen pieces were embedded in Tissue-Tek on cryostat mold and allowed to harden at −20 °C for 20 mins. Using the Microm HM 5500 Cryostat, embedded tissues were cut into sections of 12 µm thickness and dried on slides. Slides that were not used immediately were stored at −80 °C. All samples for an experiment were stored and stained in parallel. Oil red O staining of cryostat cut liver sections was as described by Mehlem et al.^51^ Briefly, sections were encircled with a Millipore ImmunoPen (402176-1EA) and incubated at room temp in ~100 µL of ORO working reagent (1.5:1) for 5 min. Slides were washed in a slide holder under running tap water for one hour. Slides were mounted with DABCO, coverslips were placed and sealed with clear nail polish. Images were taken using the EVOS® FL Auto Imaging System. Under the “Image” tab, the “Settings” was set to “Color” to capture images by light microscopy. To capture and stitch tiled images, the “Scan” tab was selected and a new tiling routine was made by selecting the “Create New Routine” button. The 20× objective was selected to capture images within a region selected by placing two “Markers” on a diagonal across the tissue section. Images were automatically stitched after acquisition. Sections were quantified using FIJI (ImageJ) software. For each image, the “Image Type” was set to RGB stack and the background was subtracted by using the “Subtract Background” feature under the “Process” menu. To measure the “Area of Lipid Droplets” in Fig. 1c, the lower threshold was set to 50 pixels and the upper threshold set to 150 pixels for each image. A ROI was established within the perimeter of the tissue section to avoid “margins” artifacts. The fraction of the total ROI area with lipid droplets was calculated using the “Area Fraction” measurement within the “Measure” feature.

### Protein extraction and western blotting

Tissues, stored at −80 °C, were homogenized with a Thomas Pestle Tissue Grinder (Size O) in freshly prepared modified RIPA buffer containing: 50 mM Tris HCl (pH 7.4), 150 mM NaCl, 5 mM EDTA, 5 mM EGTA, 2.5 mM sodium pyrophosphate, 50 mM NaF, 1 mM sodium vanadate, 20 mM β-glycerophosphate, 1 nM okadaic acid, 1 mM PMSF, 2% SDS, 0.5% sodium deoxycholate, protease (Pierce™ Protease Inhibitor Mini Tablets) and phosphatase inhibitor (Phosphatase Inhibitor Cocktail 2). Tissues were transported from the −80 °C freezer in liquid N2 and immediately homogenized on ice with ice-cold homogenization buffer (20μL/mg tissue). Homogenates were immediately centrifuged using the Eppendorf Minispin Plus at 14,100×*g* (14,000 rpm) for 10 min at 4 °C. Lipid layer was aspirated and supernatant collected, with an aliquot reserved for determining protein concentration. Sample buffer 2× was added 1:1 volume to supernatant and aliquots were stored at −80 °C. Aliquots were used only once. Electrophoresis (BioRad PowerPac HC system) was done using 40 µg of supernatant (protein concentration determined using Pierce BCA protein assay kit) at 200 V for 50 min on either a 5, 10, 12, or 15% acrylamide gel. Proteins were transferred onto nitrocellulose blots at 100 V for 50 min and blocked in BioRad Blotting-Grade Blocker (#170-6404) diluted to 5% in TBST. Antibodies were diluted and incubated according to manufacturer protocol. Pierce™ ECL Western Blotting Substrate was used to detect HRP-conjugated antibodies, bands were visualized using ImageQuant LAS 4000 and quantified with ImageJ and/or ImageQuant TL software.

### Immunostaining of liver sections with KDEL and Grp78/BiP antibodies

To carry out immunostaining of liver sections with KDEL and Grp78/BiP antibodies, mice were deeply anesthetized with isofluorane and perfused though the left ventricle with heparinized saline (0.9% NaCl containing two units of heparin/mL at a rate of 3–4 mL/min for 30 min and then with 4% formaldehyde in PBS, pH 7.4 for another 30 min as previously described^13^. Liver of mice were harvested, post-fixed in PBS containing 4% formaldehyde for 48 h at room temperature, washed with PBS and stored in PBS containing 0.01% sodium azide at 4 °C. Livers were transferred to PBS solution containing 30% (W/V) sucrose and 0.01% and stored at 4 °C until tissue sinks. Livers were then embedded in O. C. T. Compound Embedding Medium and 30 μm sections were cut using the Thermo Scientific Microtome Cryostat Microm HM 525. Sections were incubated for 1 h at room temperature on a plate shaker set at 200 rpm with 0.5% Triton x-100 in PBS (permeabilization step). To carry out immunostaining by using the mouse antibody against KDEL, liver sections were incubated for 1 h at room temperature on a plate shaker set at 200 rpm with PBS containing 0.5% Triton x-100 (permeabilization step) and then for 1 h with the M.O.M. mouse “IgG blocking reagent”. Sections were washed two times for 5 min and then incubated for 5 min in working solution of M.O.M. “diluent”. Sections were then incubated for 48 h 4 °C on plate shaker set at 200 rpm with mouse anti-KDEL antibody diluted 1:500 in M.O.M. “diluent” and then washed at room temperature on plate shaker set at 200 rpm four times for 10 min. Sections were incubated with DyLight 488-conjugated goat anti-rabbit antibodies diluted in PBST containing 0.1% Triton x-100 and 1% BSA on a plate shaker set at 200 rpm at 4 °C overnight. Sections were washed as described above and transferred to gelatin-coated microscope slides. Tissues on microscope slides were dried in the dark for 15 min before adding 1,4-diazabicyclo [2.2.2] octane (DABCO) mounting medium (40 mL containing 100 mg DABCO dissolved in 10 mL PBS with the addition of 30 mL glycerol) and mounting the coverslip. To carry out immunostaining of liver sections using rabbit antibody against Grp78/BiP we used the same immunofluorescence protocol as previously described^13^. Images of liver sections were taken using a confocal microscope (Olympus Fluoview FV1000) equipped with 20×/0.85 N.A Plan Apochromatic oil objective. All images of the same experiment were obtained with identical acquisition parameters. Images were saved as Tiff files and quantified using ImageJ. To measure abundance of KDEL across the hepatic lobule using the “Straight Line Tool” of ImageJ, three lines per image were drawn across the hepatic lobule avoiding the central vein. Pixel intensity across the straight line was measured by selecting, in the ImageJ “Analyze” menu, “Plot Profile”. The sum of the pixel intensities measured along the straight line (fluorescence intensity) was calculated and the average fluorescence intensity was derived from data obtained from three lines per image, using three images per mouse. To monitor abundance of KDEL in pericentral hepatocytes, a circle of 125 μm was placed around the center of a hepatic vein and ROIs were created around morphologically recognizable hepatocytes using the “Freehand Selections Tool” of ImageJ. The raw integrated fluorescence intensity of the selected ROIs was obtained by using the ROI Manager, and the average fluorescence intensity per hepatocyte was calculated.

### Cell culture and treatment with elevated palmitate and PE

Mouse hepatoma Hepa1–6 cells were obtained from ATCC® (#CRL-1830™) and cultured in DMEM with 10% fetal bovine serum (FBS) and 5% penicillin/streptomycin (growth medium) as previously described^46^. To treat cells with palmitate, 5 mM palmitate in 5% FFA-free BSA was prepared as previously described^52^. To make a 50 mM PE solution, 5 mg of PE was dissolved in 0.1 mL NaOH. FFA-free BSA was prepared as a 5% solution was sterile filtered. To make 5 mM solution of PE coupled to albumin, 50 μL of PE stock solution heated at 72 °C was combined with 450 μL 5% FFA-free BSA at 37 °C. To treat cells with 25 μM palmitate, the 5 mM PE solution was added directly to the medium. Detection of mycoplasma was carried using DAPI staining following an online protocol (https://projects.iq.harvard.edu/files/hlalab/files/mycoplasm-test_hla.pdf).

### Analysis of mitochondrial function and ROS

Oxygen consumption rate (OCR) of Hepa1–6 cells was monitored by the Agilent Seahorse XF Cell Mito Stress Test using the Agilent Seahorse XFe96/XF96 analyzer. Hepa1–6 cells were treated with: no additions; 200 μM palmitate; 200 μM palmitate and 25 μM PE, 200 μM palmitate and 2 mM PBA in DMEM added to growth medium for 16 h. Approximately 1 h before the assay, the cell medium was changed to Seahorse XF Base Medium containing 4 mM glucose and 1 mM pyruvate, and then cells were kept in an incubator without CO_2_ at 37 °C. For the assay, oligomycin A (4 μM), FCCP (1 μM), and antimycin/rotenone (1 uM) were added to Hepa1–6 cells from 4 mM stock solutions in DMSO, following the manufacturer instructions.

Change of Mitotracker fluorescence intensity upon addition of FCCP to monitor mitochondrial membrane potential (MMP) was measured as described previously^46^. Briefly, Hepa1–6 cells were pre-treated with and without FCCP (1 μM) in growth medium for 5 min and then MitoTracker was added at a final concentration of 200 nM from a 0.2 μM stock solution in DMSO. Cells were incubated at 37 °C for 30 min, washed twice with HBSS, and imaged with Olympus Fluoview FV1000 using 60×/1.42 N.A Plan Apochromatic oil objective. For simultaneous monitoring of MMP and ROS, Hepa1-6 cells were pre-treated at 37 °C for 20 min with 5 μM 2,7-Dichlorofluorescin diacetate by adding the dye from a 5 mM stock solution in DMSO directly to the cell growth medium. Then 200 nM MitoTracker was also added to the medium and cells were further incubated at 37 °C for 30 min, washed twice with HBSS, and transferred in HBSS containing 4 mM glucose and 1 mM pyruvate on the stage of the Olympus Fluoview FV1000.

### Quantitative RT-PCR (qRT-PCR)

Total RNA was extracted from cell cultures, after homogenizing the samples in Trizol (Life Technologies, Grand Island, NY, USA) according to the manufacturer’s instructions. The mRNA was reverse-transcribed using the High-Capacity cDNA Reverse Transcription Kit (Applied Biosystems, Foster City, CA, USA). The cDNA was amplified by quantitative RT-PCR using TaqMan Universal PCRMaster Mix (Life Technologies) according to the manufacturer’s directions. The following TaqMan assays from Life Technologies were used: ATF4 Mm00515325_g1; CHOP Mm01135937_g1; sXBP1 (Forward 5′CTGAGTCCGCAGCAGGT3′, reverse 5′TGTCAGAGTCCATGGGAAGA3′, probe FAM5′GGCCCAGTTGTCACCTCCCC3′NFQ); and the house-keeping gene ribosomal protein S2, Mm00475528_m1. Relative mRNA levels were calculated by normalizing to ribosomal protein S2 using the delta Ct method^53^.

#### Statistical analysis

All statistical analyses were performed using GraphPad Prism 6 software (GraphPad Prism, RRID: SCR_002798). Statistical significance was calculated by two-tailed unpaired *t*-test on two groups or, where indicated, by one-way ANOVA with Turkey’s multiple comparisons test on multiple groups. A value of *p* < 0.05 was considered statistically significant. The predefined criterion of the analysis has been to present all data including outliers. No test for normality was performed. Blinding was not performed. For experiments using cells, sample size was based on previous immunofluorescence experiments carried out using Hepa1–6 cells and using more than 45 cells per condition for every experiment^52,54,55^. For lipidomic analysis of murine sample size was based on our prior studies using four animals per group to determine changes in PE abundance in liver^13^.

## Supplementary information

Author Contribution

## Data Availability

The datasets generated during and/or analyzed during the current study are available from the corresponding author on reasonable request.
