# Supplementary material for: Delivery of phosphatidylethanolamine blunts stress in hepatoma cells exposed to elevated palmitate by targeting the endoplasmic reticulum
Source: Cell Death Discov. 2020 Feb 18;6:8. doi: 10.1038/s41420-020-0241-z (PMC7028721; doi:10.1038/s41420-020-0241-z)
Supplement: Supplementary file 1 — Author Contribution [file 41420_2020_241_MOESM1_ESM.pdf]

DECLARATION OF CONTRIBUTIONS TO ARTICLE

ADMC

Manuscript Number: CDDISCOVERY-19-815T

Journal Name: CELL DEATH DISCOVERY (the 'Journal')

Proposed Title of the Contribution:

Delivery of phosphatidylethanolmine blunts stress in hepatoma cells exposed to elevated palmitate by targeting the endoplasmic reticulum (the 'Contribution)

Author(s):

Marcus Trentzsch, Eugene Nyamugenda, Tiffany K. Miles, Haven Griffin, Susan Russell, Brian Koss, Kimberly A. Cooney, Kevin D. Phelan, Alan J. Tackett, Sri Iyer, Gunnar Boysen and Giulia Baldini (the 'Authors')

For all covery articles, each person named as an author in the published version must be able to show he or she has contributed substantially to the article.

Authorship credit should be based on 1) substantial contributions to conception and design, acquisition of data, or analysis and interpretation of data; 2) drafting the article or revising it critically for important intellectual content; and 3) final approval of the version to be published. Authors should meet conditions 1, 2 and 3.

Any person who cannot be shown to have made a substantial contribution to the article cannot be listed as an author in the final version. The name of any person who is deemed to have made a minor contribution can, however, appear in the Acknowledgments section of the article.

Please complete the table below to indicate the contributions of all named authors to the manuscript.

Author Full Name:	Specification of Contribution to the Manuscript:
Marcus Trentzsch	condition 1, 2 and 3
Eugene Nyamugenda	condition 1,2 and 3
Tiffany K.Miles	condition 1, 2 and 3
Haven Griffin	condition 1 , 2 and 3
Susan Russell	condition 1,2 and 3
Brian Koss	condition 1, 2 and 3
Kimberly A. Cooney	condition 1, 2 and 3
Kevin D. Phelan	condition 1, 2 and 3
Alan J. Tackett	condition 1, 2 and 3
Sri Iyer	condition 1, 2 and 3
Gunnar Boysen	condition 1,2 and 3
Giulia Baldini	condition 1,2 and 3

Please complete the table below to indicate the contributions of all named authors to the figures.

Figure 1:
EN, TKM, HG, SR, KAC, GBaldini

Figure 2:
MT, EN, GBoysen, GBaldini

Figure 3:
S.I., EN, GBaldini

Figure 4:
E.N., GBaldini

Figure 5:
E. N., GBaldini

Figure 6:
B. K., A.J.T., GBaldini

Signed for and on behalf of the Author(s):	Print Name:	Date:
Giulia Baldini	Giulia Baldini	Dec 4, 2019
